# Population Dynamics and Flight Phenology Model of Codling Moth Differ between Commercial and Abandoned Apple Orchard Ecosystems

**DOI:** 10.3389/fphys.2016.00408

**Published:** 2016-09-22

**Authors:** Neelendra K. Joshi, Edwin G. Rajotte, Kusum J. Naithani, Greg Krawczyk, Larry A. Hull

**Affiliations:** ^1^Department of Entomology, Pennsylvania State University, University ParkPA, USA; ^2^Fruit Research and Extension Center, Entomology, Pennsylvania State UniversityBiglerville, PA, USA; ^3^Department of Biological Sciences, University of ArkansasFayetteville, AR, USA

**Keywords:** codling moth, phenology, flight model, commercial orchards, abandoned orchards, insecticide resistance

## Abstract

Apple orchard management practices may affect development and phenology of arthropod pests, such as the codling moth (CM), *Cydia pomonella* (L.) (Lepidoptera: Tortricidae), which is a serious internal fruit-feeding pest of apples worldwide. Estimating population dynamics and accurately predicting the timing of CM development and phenology events (for instance, adult flight, and egg-hatch) allows growers to understand and control local populations of CM. Studies were conducted to compare the CM flight phenology in commercial and abandoned apple orchard ecosystems using a logistic function model based on degree-days accumulation. The flight models for these orchards were derived from the cumulative percent moth capture using two types of commercially available CM lure baited traps. Models from both types of orchards were also compared to another model known as PETE (prediction extension timing estimator) that was developed in 1970s to predict life cycle events for many fruit pests including CM across different fruit growing regions of the United States. We found that the flight phenology of CM was significantly different in commercial and abandoned orchards. CM male flight patterns for first and second generations as predicted by the constrained and unconstrained PCM (Pennsylvania Codling Moth) models in commercial and abandoned orchards were different than the flight patterns predicted by the currently used CM model (i.e., PETE model). In commercial orchards, during the first and second generations, the PCM unconstrained model predicted delays in moth emergence compared to current model. In addition, the flight patterns of females were different between commercial and abandoned orchards. Such differences in CM flight phenology between commercial and abandoned orchard ecosystems suggest potential impact of orchard environment and crop management practices on CM biology.

## Introduction

Codling moth (CM), *Cydia pomonella* (L.) (Lepidoptera: Tortricidae), is an economically important deciduous orchard pest in the northeastern (Dean, 1989) as well as in the western U.S. apple growing regions (Beers et al., 1993). In Pennsylvania, it has been a major pest in all apple growing regions (Hodgkiss et al., 1934; Worthley and Marston, 1935) for more than 70 years, and began to re-emerge as a serious pest in the late 1990's (Hull et al., 2001).

Pest management approaches including different insecticides (Hull et al., 2009a,b) and mating disruption products (Joshi et al., 2008; Bohnenblust et al., 2011a) are used for controlling both adults and larvae (such as CM and the oriental fruit moth [OFM], *Grapholita molesta* [Busck]) in commercial apple orchards in this region. At present, growers mainly time insecticide sprays against CM based on a phenology model (PETE - prediction extension timing estimator-henceforth referred as “PETE model”) developed initially in Michigan in the 1970's (Riedl et al., 1976; Welch et al., 1978) using insecticide susceptible populations in non-commercial/abandoned apple orchards. Since then CM has developed resistance to various insecticides in commercial apple orchards around the world (Riedl et al., 1986; Bush et al., 1993; Varela et al., 1993; Knight et al., 1994; Dunley and Welter, 2000; Mota-Sanchez et al., 2008). In Pennsylvania apple orchards, widespread insecticide resistance has been observed in CM populations over the past 15–20 years (Krawczyk and Hull, 2005). These insecticide-resistant CM populations may develop at different growth rates as reported elsewhere (Lue, 2005), and it is very likely that CM phenological predictions are different from the currently used PETE model for many Pennsylvania apple orchards, and may be in other apple growing regions. Lue (2005) reported that insecticide-resistant CM populations may develop at different growth rates, and may have different flight emergence/spring emergence patterns than the normal (i.e., non-resistant) populations. Such differences may result in mistimed insecticide applications, reduced pesticide effectiveness, and pesticide overuse in commercial orchards due to the poor predictive performance of the model developed from non-resistant populations. Predictive models developed from a normal (insecticide susceptible) population of CM should include adjustments to accommodate life history changes, such as local ecotype evolution and pesticide resistance.

Population dynamics and seasonal phenology of insect pests (i.e., CM) is generally influenced by several biotic and abiotic factors. Insects are poikilotherms, as their body temperature is regulated by the ambient temperature. Effect of temperature among other factors on the development and seasonal phenology of various insect pest species (Messenger and Flitters, 1958; Allen, 1976; Akers and Nielsen, 1984; Wagner et al., 1984; Damos and Savopoulou-Soultani, 2008) including CM (Riedl et al., 1976; Welch et al., 1978; Jones et al., 2013; Kumar et al., 2015) had been widely studied in the past. Such findings have also been widely used in modeling phenology and predicting development or life cycle events of respective insect species over time (Damos et al., 2011), and have been applied extensively in integrated pest management (IPM) programs of various tree fruit pests (Riedl et al., 1976; Welch et al., 1978; Croft et al., 1980; Pruess, 1983; Knight, 2007; Damos, 2009; Damos and Savopoulou-Soultani, 2010). Although temperature is known as the major factor influencing CM development and phenology, other environmental factors such as the effects of crop management and orchard practices may also have influence on CM biological parameters, and their role cannot be underestimated.

Considering the potential impacts of orchard environment and pest management practices on the developmental biology and phenology of CM, this study was designed to address the following questions: (1) Is CM flight phenology in commercial and abandoned orchards different than those predicted by the currently used PETE model (developed in 1970s); (2) does the flight phenology of male and female CM differ in commercial and abandoned orchard ecosystems; and (3) how do the re-specified models perform in other commercial orchards during 2007, 2008, and 2009.

## Materials and methods

Field studies were conducted to study the flight bionomics of adult CM in four commercial and four abandoned apple orchards, each with an infestation history of CM during 2007–2008. All orchards were located in Adams County, PA, USA.

### Description of abandoned and commercial apple orchard ecosystems

All test orchards were in Adams County, PA and were planted with mixed (more than two) cultivars. In abandoned orchards, the tree height was ≈ 7–8 m, and the trees were ≈45+ years old. In commercial orchards, the tree height was ≈ 2.5–3.5 m, and the trees were ≈20+ years old. All abandoned orchards had been neglected for at least 7–15+ years, while the commercial orchards were regularly managed with conventional insecticide programs (Anonymous, 2012). Abandoned orchard sites were at the following locations: Site 1- (39°.49. 1830° N, 77°.22. 3138° W), Site 2- (39°.58. 5260° N, 77°.14. 6138° W), Site 3- (39°.59. 1289° N, 77°.14. 4126° W), and Site 4- (39°.59.7791°N, 77°.14.9361° W), and the commercial sites were at the following locations: Site 1- (39°.50. 2999° N, 77°.11. 4278° W), Site 2- (39°.58. 4723° N, 77°.14. 2756° W), Site 3- (39°.59. 1694° N, 77°.14. 9140° W), and Site 4- (40°. 0. 3478° N, 77°.14. 7020° W). The distance between abandoned and commercial orchards at each site was 1.5–2.5 km, and the distance between the orchards located at Site 1 and the orchards located at Site 4 was ~40 km.

### Monitoring of adult codling moth populations

Two types of commercially available CM sex pheromone monitoring lures from Trécé Inc. (Adair, OK, USA, 74330), viz. CM Long-Life™ L2™ (hereafter referred to as CM L2) (3.5 mg of (*E, E*)-8,10-dodecadien-1-ol in gray halobutyl septum) and Pherocon® CM-DA Combo™ (mixture of 3.0 mg of (*E, E*)-8, 10-dodecadien-1-ol and 3.0 mg of ethyl (2*E*, 4*Z*)-2, 4 decadienoate in gray halobutyl septum) were used in the study orchards to determine the flight bionomics of CM throughout the growing season each year. The CM DA Combo lure (hereafter referred as CM DAC) acts as an attractant to both sexes of CM, while the CM L2 lure attracts only male moths (Light et al., 2001; Knight and Light, 2005; Joshi et al., 2011a; Bohnenblust et al., 2011b).

### Placements of traps on trees and traps maintenance

In both types of orchard ecosystems, both lure types were placed in Large Plastic Delta traps (Trécé Pherocon VI®, Adair, OK) attached to bamboo poles (≈2–2.5 m in length) placed in the upper 2/3rd of the tree canopy. Four traps (2 traps/ lure type) were placed in all commercial and abandoned orchards, except the orchards at Site 2, where six traps (3 traps/lure type) were placed. Each trap was placed at ≈90–100 m apart in a row and ≈20–25 m from the border. The trap-tree rows were spaced at least 70 m apart from each other. The traps were placed in all orchards on 23 April and 20 April in 2007 and 2008, respectively, and trap monitoring started approximately 1 week after trap placement. The traps were checked twice a week for moth capture. The effect of trap location and lure type on moth capture was minimized by rotating the position of each trap (in clock-wise manner) to the next trap location within each trap-tree row at every observation period. All captured moths were sexed. The sticky liners of all traps were changed at least every 2 weeks or as needed. Lures were changed after 8 weeks in all orchards, and in both years, all the traps were checked until the end of the season (i.e., mid-October).

### Statistical analysis and model development

The percent cumulative moth capture of CM for each orchard was determined and plotted over the accumulated degree-days from biofix (i.e., first sustained moth capture) for each study site to construct models for predicting CM flight. In both years, the biofix was established according to emergence of male moths in all commercial and abandoned orchard ecosystems.

The degree-days for the entire study period were calculated during the moth trapping period for each study site from the daily maximum and minimum temperatures (maximum temperature + minimum temperature/2). High resolution temperature data (<1 km) were obtained from ZedX, Inc. (Bellefonte, PA, 16823). For degree-day calculations, threshold temperatures of 10°C (50°F) and 31°C (88°F) were used as lower and upper thresholds, respectively (Glenn, 1922a,b).

The mean cumulative percent moth capture of CM (after the biofix date) for each generation was used in constructing the logistic flight models for the respective orchards. (Neter and Wasserman, 1974; Knight, 2007):

(1) $$\begin{array}{l} E\left(Y\right)=e^{\left(\beta_{0}+{\beta_{1}}^{*}X\right)}/\left(1+e^{\left(\beta_{0}+{\beta_{1}}^{*}X\right)}\right) \end{array}$$

where Y is the proportion of the event completed (mean cumulative percent moth capture), X is the cumulative degree-days from biofix date, β_0_ = Intercept and β_1_ = Slope. The β_0_ and β_1_ parameters of the logistic equation were estimated with linear regression by first transforming the proportions (p) into logits (*p*′) as follows (Neter and Wasserman, 1974; Knight, 2007):

(2) $$\begin{array}{l} p\prime =Log_{e}\left[p/\left(1-p\right)\right] \end{array}$$

Based on the duration of the first and second generation CM flight curves, the following two types of modeling approaches were tested, and compared with the currently used PETE model for predicting CM flight phenology:

- In the constrained model, the degree-day cutoff (i.e., completion) for the first or second generation flights of CM from the date of biofix (in terms of accumulated DD) was assumed to be identical to that utilized by the current model (i.e., PETE). In both years, the degree-day cutoffs for the constrained models for both the first and second generation flights were assumed as ~455–465 DD and ~1055–1060 DD, respectively. This model is termed constrained PCM.
- In the unconstrained model, the degree-day cutoff for the first or second generation flights of CM from biofix (in terms of accumulated DD) was based on actual moth captures, i.e., end of first or second generation flight as determined from the mean seasonal moth capture graphs (or in other words, complete or near complete shut-down of moth capture in the traps or the lowest trap capture date near the estimated completion date of both generations). In both years, the degree-day cutoffs for this model for both the first and second generation flights were determined to be ~530–550 DD and ~1175–1195 DD, respectively. This model is termed unconstrained PCM.

### Model comparisons and evaluation

The logistic model (for the current model) for CM flight/adult emergence was derived from the tabular values for the PETE model (Brunner and Hoyt, 1982). An analysis of covariance (ANCOVA) was used to determine differences between the slopes and intercepts of the logistic regression equations for both flight models (i.e., constrained and unconstrained PCM) in both commercial and abandoned orchards. The PCM models were compared with the original logistic PETE model for CM flight patterns (in terms of adult emergence) in both types of orchard ecosystems.

Based on the mean seasonal moth capture analyses for both years, the unconstrained PCM model was found to be more representative of CM flight patterns in abandoned and commercial apple orchards in Pennsylvania. Therefore, the PCM unconstrained model was further evaluated for its performance in three different commercial orchards for 3 years (2007, 2008, and 2009). The commercial orchards where the model performance was evaluated were located in Adams County, PA, USA, and received only conventional insecticide applications. The daily minimum and maximum temperatures for these orchards were obtained from the Fruit Research and Extension Center in Biglerville, PA. The R software (R Development Core Team, 2005; ISBN 3-900051-07-0) was used to perform all data analyses including data transformation and logistic modeling, and graphs were generated in SigmaPlot® 11 (Systat Software, Inc., Chicago, IL USA).

## Results

### General seasonal flight patterns of CM in commercial and abandoned orchards

**Figure 1:**
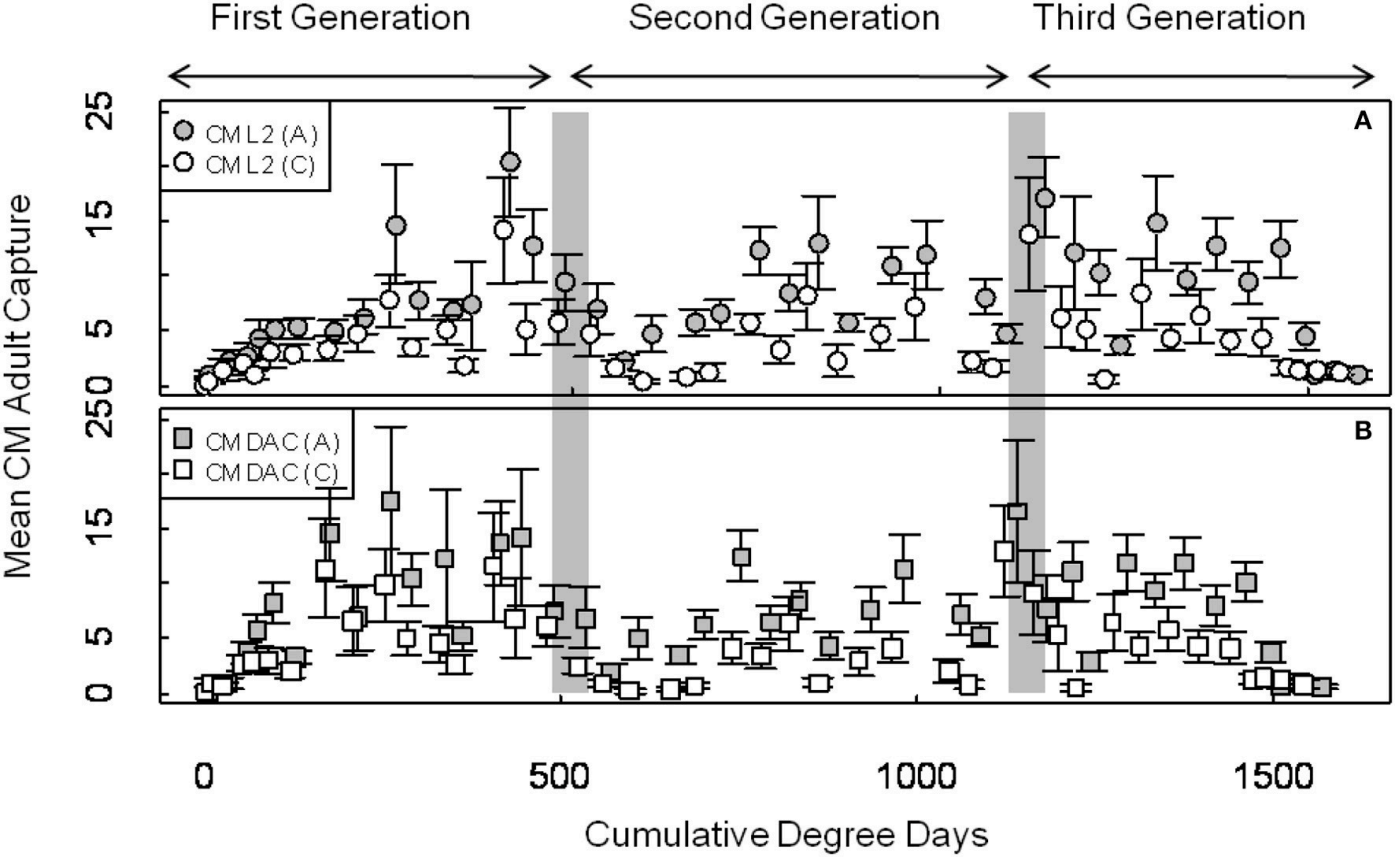
**Seasonal flight patterns in terms of mean seasonal moth capture (±SEM) per trap (first and second generation) using CM L2 (A) and CM DAC (B) lures in abandoned (A) and commercial (C) apple orchards in Pennsylvania in 2007**. A light gray band on the graph indicates ranges of PETE-CM model/constrained model degree days cutoff for the first and second generations of CM. Degree days (on degree Celsius scale) are cumulative degree days started from the respective date of biofix in both types of orchards.

**Figure 2:**
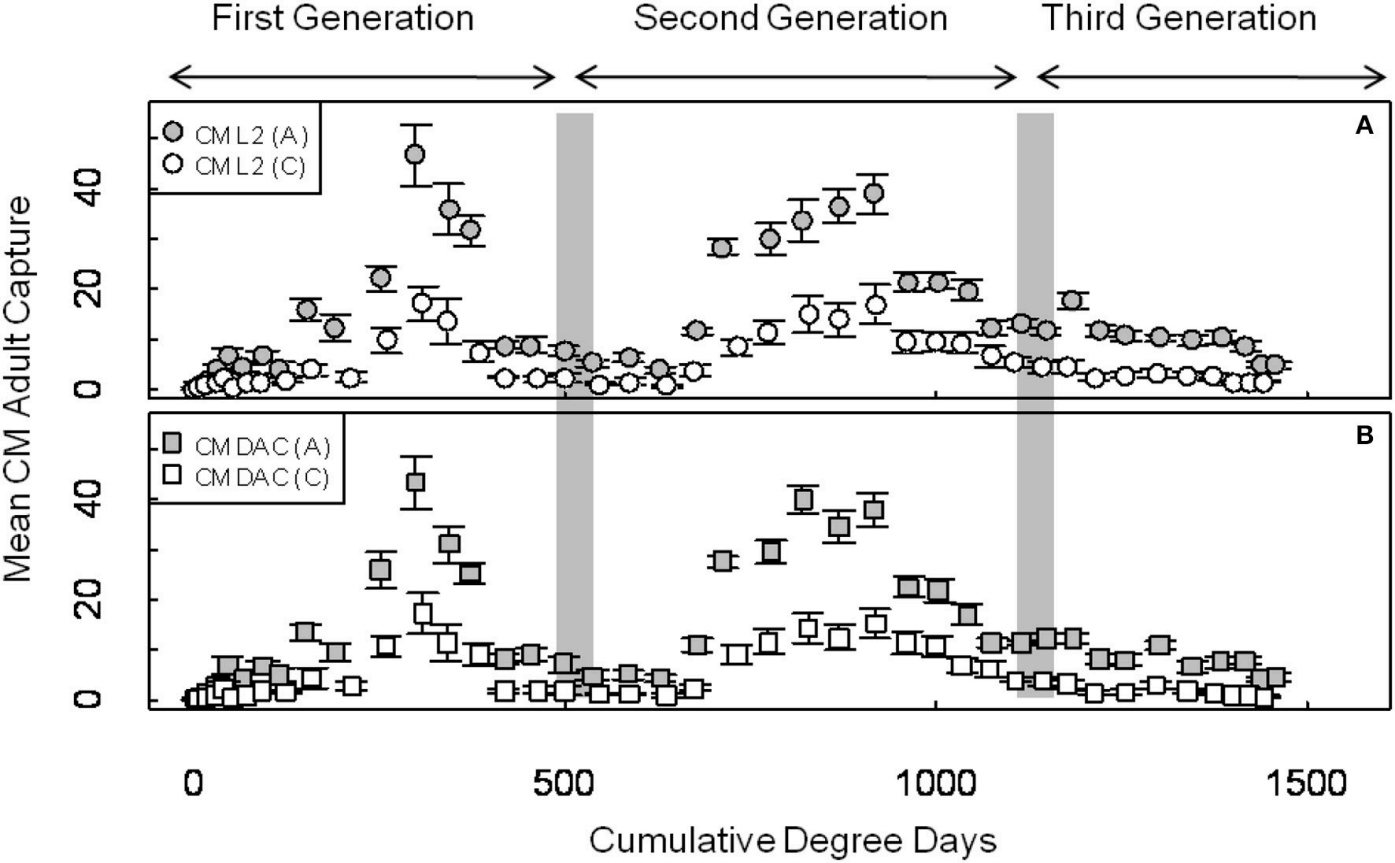
**Seasonal flight patterns in terms of mean seasonal moth capture (±SEM) per trap (first and second generation) using CM L2 (A) and CM DAC (B) lures in abandoned (A) and commercial (C) apple orchards in Pennsylvania in 2008**. A light gray band on the graph indicates range of PETE-CM model/constrained model degree days cutoff for the first and second generations of CM. Degree days (on degree Celsius scale) are cumulative degree days started from the respective date of biofix in both types of orchards.

Mean seasonal adult moth captures in abandoned and commercial orchards were consistently high in both years, and populations of CM were generally higher in abandoned orchards than in commercial orchards (Figures 1, 2). In 2007, the mean seasonal moth capture in CM L2 baited traps (Figure 1A) and CM DAC baited traps (Figure 1B) showed a distinct bimodal emergence pattern during the first generation. However, in 2008 (Figures 2A,B), the bimodal emergence peaks of first generation were not as distinct as in 2007. During both years at the end of first and second generation flights, the lowest mean seasonal moth capture (degree day cutoff) occurred around ~550 DD and ~1230 DD, respectively (Figures 1, 2). In both years, moth capture in CM L2 and CM DAC baited traps showed very similar seasonal patterns for both abandoned and commercial orchard ecosystems (Figures 1, 2). Also, a partial third generation was recorded in both years (Figures 1, 2). In both orchard types, moth flight in both years declined rapidly after ~1325 DD with the last moths captured at ~1525 DD (Figures 1, 2).

### Codling moth male flight: PCM model development in abandoned and commercial orchards

**Figure 3:**
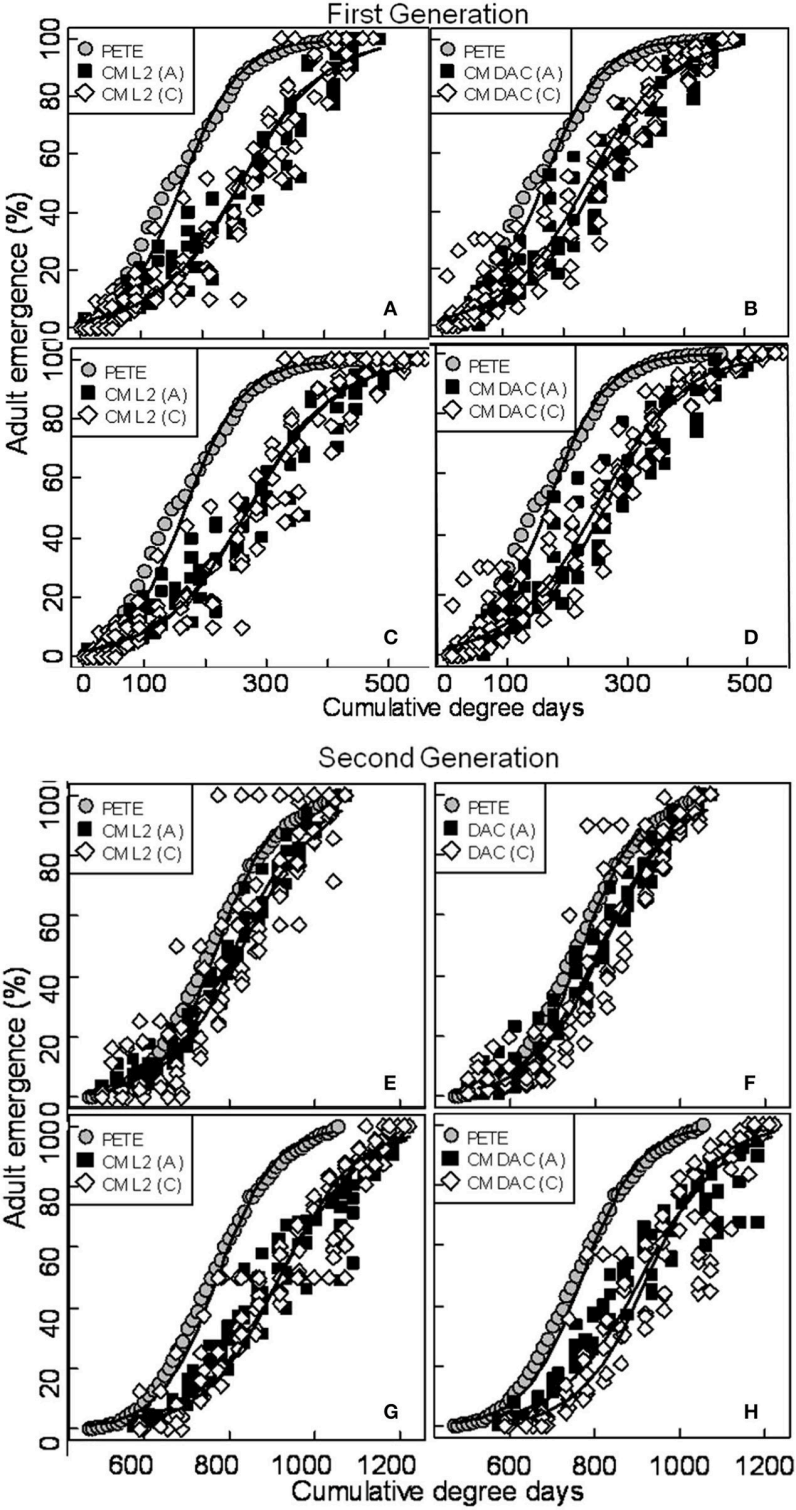
**Prediction of seasonal male flight patterns of codling moth in terms of cumulative percent capture of adults [first (A,B,C,D) and second generation (E,F,G,H)] using CM L2 and CM DAC lures in abandoned (A) and commercial (C) apple orchards by constrained, unconstrained and PETE CM logistic models**. A comparison of constrained **(A,B,E,F)** and unconstrained **(C,D,G,H)** PCM logistic flight models with PETE-CM model is shown. The models were developed using moth capture data collected during 2007 and 2008. Degree days (on degree Celsius scale) are cumulative degree days started from the respective date of biofix in both types of orchards. In each graph, symbols represent observed data and solid lines represent models.

When moth capture data using the CM L2 lures were combined over both years, the flight curves generated by both constrained (Figures 3A,E) and unconstrained (Figures 3C,G) PCM models were significantly different from the predicted emergence curves generated by the current PETE model for both generations of CM and for both orchard ecosystems (*P* = 0.000; Tables 1, 2). In abandoned and commercial orchards, the PCM constrained (Figures 3A,E) and unconstrained (Figures 3C,G) models showed differences in the prediction of CM flight curves for both first and second generations at intercept (*P* = 0.000; Tables 1, 2), but not at slope (*P* > 0.05; Tables 1, 2).

**Table 1:** **Model parameters of logistic PCM models and PETE model for codling moth (first and second generation) flight in commercial and abandoned orchards**.

**Lure (orchard type)**	**Intercept**	***SE***	**Slope**	***SE***	**Adjusted *R*^2^**
**CONSTRAINED MODEL**					
**First generation**					
CM L2 (A)	−3.912	0.113	0.008	0.0003	0.89
CM L2 (C)	−3.755	0.129	0.008	0.0003	0.87
PETE	−3.517	0.133	0.012	0.0003	0.98
CM DAC (A)	−3.987	0.130	0.009	0.0003	0.89
CM DAC (C)	−3.513	0.125	0.008	0.0003	0.88
**Second generation**					
CM L2 (A)	−9.507	0.204	0.007	0.0002	0.95
CM L2 (C)	−9.439	0.471	0.006	0.0003	0.81
PETE	−11.391	0.199	0.008	0.0001	0.99
CM DAC (A)	−9.756	0.243	0.007	0.0002	0.94
CM DAC (C)	−9.930	0.416	0.007	0.0003	0.84
**UNCONSTRAINED MODEL**					
**First generation**					
CM L2 (A)	−3.954	0.106	0.008	0.0002	0.92
CM L2 (C)	−3.799	0.121	0.008	0.0003	0.90
PETE	−3.517	0.133	0.012	0.0003	0.98
CM DAC (A)	−3.978	0.122	0.008	0.0003	0.91
CM DAC (C)	−3.508	0.107	0.008	0.0002	0.92
**Second generation**					
CM L2 (A)	−9.303	0.206	0.006	0.0001	0.95
CM L2 (C)	−10.290	0.391	0.006	0.0002	0.87
PETE	−11.391	0.199	0.008	0.0001	0.99
CM DAC (A)	−9.392	0.255	0.006	0.0002	0.92
CM DAC (C)	−11.692	0.459	0.007	0.0003	0.87

*(A), Abandoned orchard; (C), Commercial orchard*.

*Cumulative moth capture data was derived from moth capture using CM L2 and CM DAC lures in commercial and abandoned orchards during 2007 and 2008*.

**Table 2:** **Statistical details (ANCOVA ***P***-values) of codling moth models comparison in commercial and abandoned orchard ecosystems**.

**Lure (orchard type)**	**PCM constrained model**				**PCM unconstrained model**			
	**First generation**		**Second generation**		**First generation**		**Second generation**	
	**Intercept**	**Slope**	**Intercept**	**Slope**	**Intercept**	**Slope**	**Intercept**	**Slope**
CM L2 (A)								
PETE	0.000^*^	0.000^*^	0.000^*^	0.000^*^	0.000^*^	0.000^*^	0.000^*^	0.000^*^
CM L2 (C)								
PETE	0.000^*^	0.000^*^	0.000^*^	0.000^*^	0.000^*^	0.000^*^	0.000^*^	0.000^*^
CM L2 (A)								
CM L2 (C)	0.000^*^	0.627	0.000^*^	0.096	0.000^*^	0.724	0.000^*^	0.823
CM DAC (A)								
PETE	0.000^*^	0.000^*^	0.000^*^	0.000^*^	0.000^*^	0.000^*^	0.000^*^	0.000^*^
CM DAC (C)								
PETE	0.000^*^	0.000^*^	0.000^*^	0.000^*^	0.000^*^	0.000^*^	0.000^*^	0.000^*^
CM DAC (A)								
CM DAC (C)	0.000^*^	0.009^*^	0.000^*^	0.313	0.000^*^	0.013^*^	0.000^*^	0.046^*^
CM DAC (A)								
CM L2 (A)	0.000^*^	0.352	0.000^*^	0.959	0.000^*^	0.354	0.000^*^	0.417
CM DAC (C)								
CM L2 (C)	0.000^*^	0.002^*^	0.000^*^	0.606	0.000^*^	0.002^*^	0.000^*^	0.285

*(A), Abandoned orchard; (C), Commercial orchard*.

*An asterisk denotes a significant difference at the 0.05 level*.

*Model parameters of these CM models are given in the Table 1*.

In abandoned as well as commercial orchards, the PCM models (both constrained and unconstrained) developed from the data (both years combined data) on moth capture using CM DAC lure baited traps showed significant differences in CM flight curves at slope and intercept for both the first (Figures 3B,F) and second (Figures 3D,H) generations from the PETE model (*P* = 0.000; Tables 1, 2). The PCM constrained model showed that the flight curves in abandoned orchards vs. commercial orchards were different at intercept and slope (*P* < 0.05) for the first generation and only at intercept for the second generation (*P* < 0.05; Tables 1, 2). In a similar comparison of CM flight curves in commercial and abandoned orchards, the PCM unconstrained model showed significant differences at both intercept and slope of CM flight for both generations (Tables 1, 2).

### Comparison of models developed from moth capture in CM L2 and CM DAC lures in abandoned and commercial orchards

In abandoned orchards, the constrained PCM flight model curves (developed from moth capture using CM L2 and CM DAC lure baited traps) showed differences in intercept for the first and second generation, but not slope (Figures 3A,B,E,F; Tables 1, 2). The unconstrained model approach also showed similar results (Tables 1, 2; Figures 3C,D,G,H). In commercial orchards, both constrained and unconstrained PCM models showed differences for both slope and intercept of the first generation and only intercept of the second generation (*P* < 0.05; Tables 1, 2; Figure 3).

### Female moth capture and flight model

**Figure 4:**
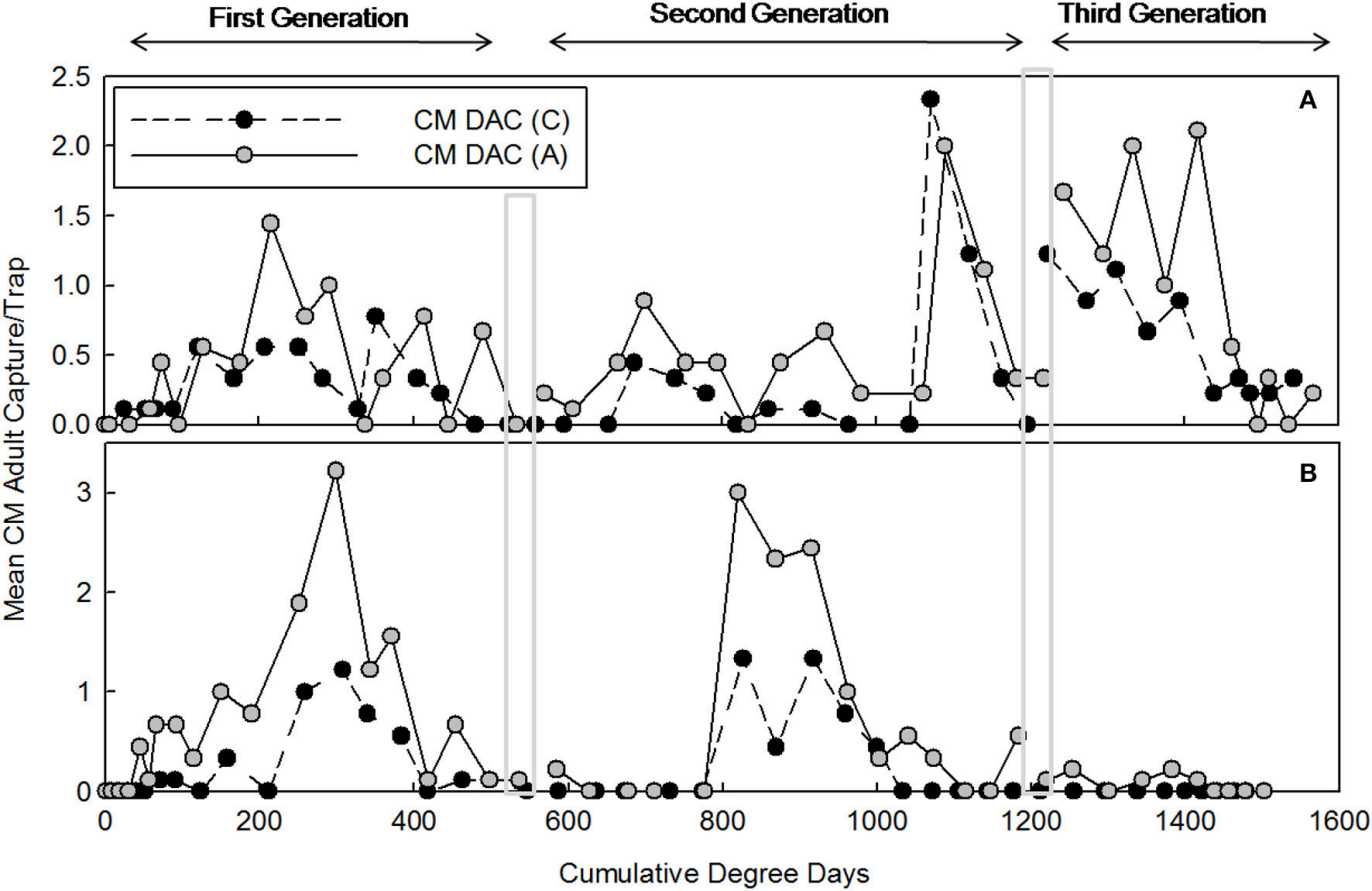
**Seasonal female codling moth flight patterns in terms of mean seasonal moth capture (±SEM) per trap (first and second generation) CM DAC lure in abandoned (A) and commercial (C) apple orchards in Pennsylvania in 2007 (A) 2008 (B)**. A light gray band on the graph indicates range of degree days cutoff for the first and second generations of CM. Degree days (on degree Celsius scale) are cumulative degree days started from the respective date of biofix in both types of orchards.

**Figure 5:**
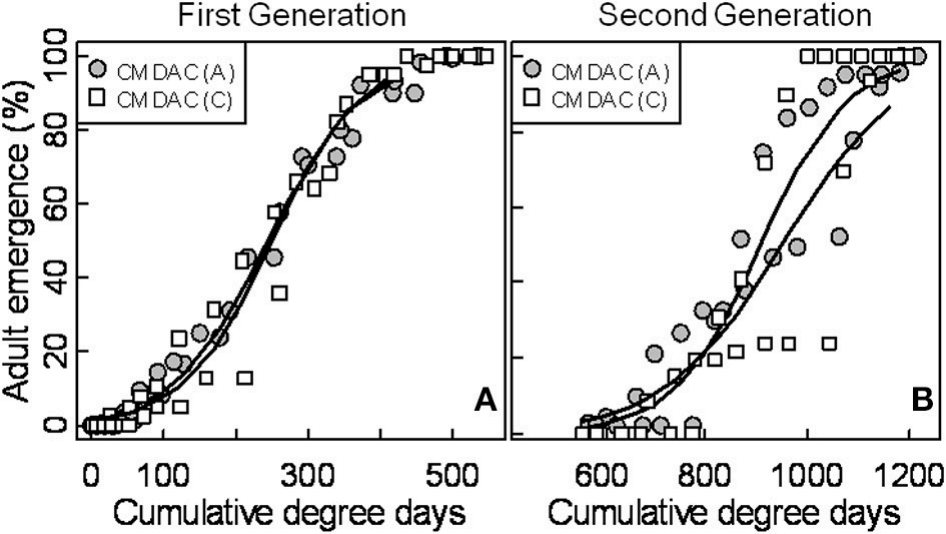
**Seasonal flight patterns of female codling moth in terms of cumulative percent capture of adults [first (A) and second generation (B)] using CM DAC lures in abandoned (A) and commercial (C) apple orchards in Pennsylvania**. The PCM female flight model was developed using moth capture data collected during 2007 and 2008. Degree days (on degree Celsius scale) are cumulative degree days started from the respective date of biofix in both types of orchards. In each graph, symbols represent observed data and solid lines represent models.

Mean seasonal female moth captures using CM DAC baited traps in abandoned and commercial orchards were consistently low in both years (Figures 4A,B). A female flight prediction model was developed from the percent cumulative female moth capture in CM DAC lure baited traps (Figures 5A,B). When the female flight curves of abandoned orchards were compared with the female flight curves of commercial orchards, the model showed significant differences in intercept for the flight curves of both generations (Table 3). However, these curves were not different in slope (Table 3).

**Table 3:** **Codling moth (first and second generation female) flight in commercial and abandoned orchards using CM DAC lures**.

**Lure (orchard type)**	**Intercept**	***SE***	**Slope**	***SE***	**ANCOVA *P*-values**		
					**Adjusted *R*^2^**	**Slope**	**Intercept**
**FIRST GENERATION**							
CM DAC (A)	−3.771	0.192	0.016	0.0007	0.95		
CM DAC (C)	−4.129	0.240	0.017	0.0009	0.94	0.000^*^	0.500
**SECOND GENERATION**							
CM DAC (A)	−10.730	0.836	0.012	0.0009	0.85		
CM DAC (C)	−8.188	2.160	0.009	0.0002	0.48	0.000^*^	0.338

*(A), Abandoned orchard; (C), Commercial orchard*.

*An asterisk denotes a significant difference at the 0.05 level*.

*Model parameters of logistic PCM model developed from 2007 and 2008 datasets and ANCOVA P-values*.

### PCM (male) flight model evaluation (predicted vs. observed) and its comparative validation in commercial orchards

**Figure 6:**
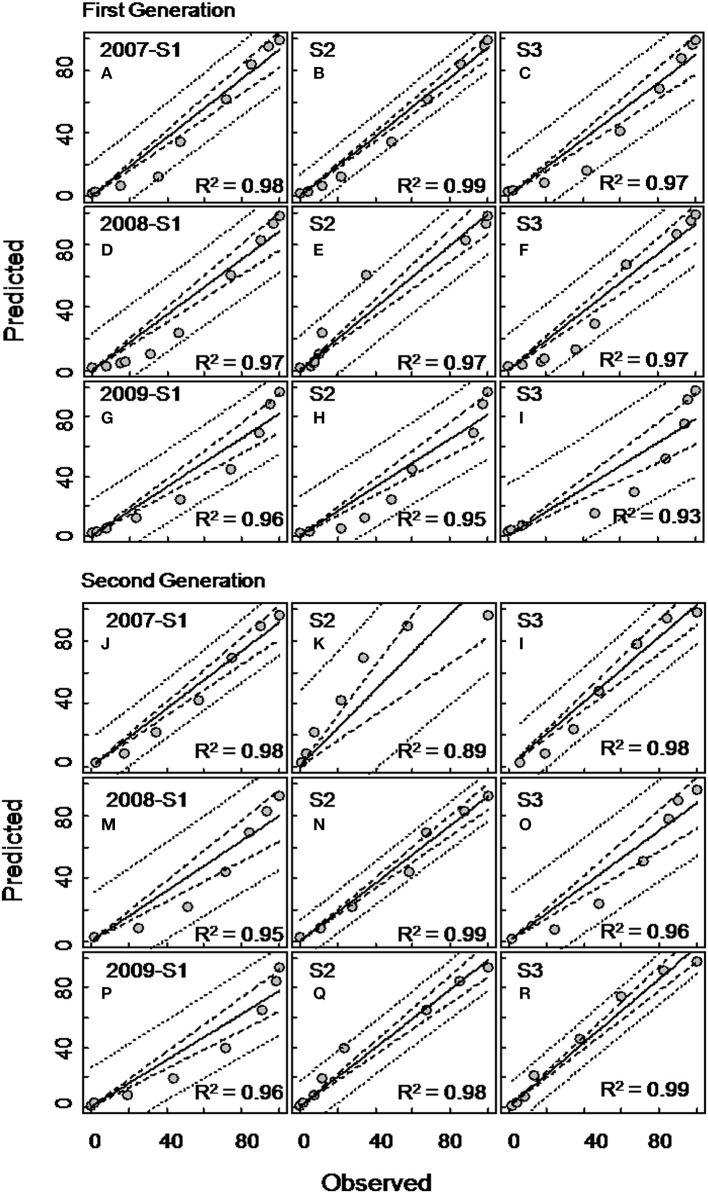
**Evaluation of the unconstrained PCM model (developed from moth captures in CM L2 lures baited traps) for codling moth flight prediction for the first (A–I) and second (J–R) generations in three different apple orchards (sites) in Adams County, Pennsylvania during 2007, 2008, and 2009**. Each graph of Site 1 (S1), Site 2 (S2), and Site 3 (S3) represents flight patterns in terms of percent cumulative moth capture using commercial lures (combined moth capture in CM L2 and CM DAC baited traps). In each graph, the inner dotted band represents 95% confidence interval and the outer dotted band represents prediction interval.

The ANCOVA analysis showed that the flight predictions from the PCM unconstrained model representing the extended moth emergence pattern was significantly different from the PETE and constrained models (Tables 1, 2). Further, the mean moth capture analysis (Figures 1, 2) showed that the PCM unconstrained model was more representative of the extended moth emergence patterns than the constrained/PETE models for PA apple orchards. Therefore, the PCM unconstrained model was further evaluated for its prediction performance and validated against 3 years of moth capture/emergence data from three different orchards in Adams County, PA. The PCM unconstrained model had the most accurate predictions of moth emergence in all three orchard sites across all 3 years (*R*^2^ > 0.95; Figure 6). The observed moth capture/emergence data (cumulative moth capture from traps baited with CM L2 and CM DAC lures) for both generations in all orchards and years fell within the 95% prediction interval range/bands of the PCM unconstrained model predictions (Figure 6).

For the commercial orchard data in 2007/2008, the newly developed PCM unconstrained model (generated from moth capture data with either CM L2 or CM DAC lure baited traps) predicted a delay in the flight emergence pattern of male adults of ~30–55 DD at the 10 percent cumulative moth emergence period during the first generation when compared to the predicted moth emergence pattern generated by the CM PETE model (Figures 3C,D). When further compared to the PETE model, the PCM unconstrained model also showed a delayed emergence of moths by ~100–125 DD and ~120–130 DD at 50 and 90 percent cumulative moth emergence level, respectively (Figures 3C,D). During the second generation of CM in commercial orchards, the unconstrained model (irrespective of lure types) showed a delayed emergence by ~125–140 DD at 10 percent cumulative moth emergence as compared to the predicted moth emergence from the CM PETE model (Figures 3G,H). Again, when compared to the PETE model, the PCM unconstrained model also showed a delayed emergence of moths by ~160–165 DD and ~150–160 DD at 50 and 90 percent cumulative moth emergence levels, respectively (Figures 3G,H).

## Discussion

In these studies we found that the currently used CM PETE model was inadequate when predicting CM adult flight in apple orchards of Pennsylvania. We propose a new model, PCM, which more accurately reflects CM flight in Pennsylvania and perhaps elsewhere.

Codling moth flight patterns in both commercial and abandoned orchard types were also different from those predicted by the PETE model. During both years, CM adult populations (in terms of mean seasonal moth capture) were significantly higher in abandoned orchards than nearby commercially managed orchards. The higher populations in abandoned orchards were most likely due to the absence of chemical insecticide programs in these orchards over the previous 5–10 years. Differences in CM abundance between these two orchard ecosystems could also be due to other factors, such as variation in tree canopy size and landscape characteristics. Also, an extended emergence/flight period for male CM was observed for both generations. In both types of orchard ecosystems, adult CM showed a bimodal emergence pattern during their first generation flight and a unimodal emergence pattern during their second generation flight. The delayed or extended emergence/flight pattern during the first generation is likely to cause subsequent delays during the moth flight of the second generation. Due to this extended emergence patterns of male CM, the completion of first and second generation flight (as showed by the mean seasonal moth capture) was delayed by ~95 and ~150 DD, respectively, from the predictions generated by the PETE model. The asymmetric emergence patterns (i.e., extended emergence of moths during both generations) of CM could be due to variability associated with diapause induction in CM larvae (Steinberg et al., 1988). A certain proportion of a CM population from each generation enters diapause (Brown et al., 1979), and the full-grown fifth instar CM larvae over-winter in a silken cocoon (Shelford, 1927). The overwintering CM larvae complete their development and life cycle upon arrival of favorable environmental conditions, primarily during the following spring season (Geier, 1963). However, adults from these diapausing larvae do not emerge together (as a cohort) at the onset of the spring season of the next year, but they emerge at different timings (Brown et al., 1979; Steinberg et al., 1988), resulting in differences in the post-diapause spring emergence timings/patterns.

In this study, when the cumulative moth capture data were fit to either a constrained and unconstrained modeling approach, both PCM models conclusively showed a delay in the emergence/flight of moths (irrespective of monitoring lure types and study year) compared to the PETE model. For instance, during the first generation, PCM unconstrained model predicted delays in moth emergence (compared to the PETE model) by ~30–55 DD, ~100–125 DD, and ~120–130 DD at 10, 50, and 90 percent cumulative moth emergence levels, respectively, in commercial orchards. Similarly, delays in moth emergence were also recorded during the second generation in commercial orchards. In both types of orchard ecosystems, the PCM models showed a delayed emergence of CM as compared to model predictions from PETE. Knight (2007) reported that the cumulative flight curves for local populations of first generation CM in Washington State were delayed and different from those reported by the PETE model in Washington. Such differences in the model prediction could be due to many factors including continual use of similar insecticidal chemistries over the last 30–40 years, which can cause resistance in CM populations.

Apple production and pest management programs in the U.S. have changed significantly during the last 30–40 years. The intensive applications of insecticides sprayed onto fruit orchards have caused some CM populations to develop resistance to a number of insecticides (Riedl et al., 1986; Bush et al., 1993; Varela et al., 1993; Knight et al., 1994; Dunley and Welter, 2000; Mota-Sanchez et al., 2008). The PETE multi-species pest complex model (Welch et al., 1978) for CM phenology, which is structured on the distributed delay methods of Manetsch and Park (1974) was developed initially in Michigan in the 1970's for insecticide susceptible (normal) populations in abandoned orchards. Pesticide application timings based on the PETE model have been successful in managing susceptible populations of CM (Riedl et al., 1976; Welch et al., 1978), as the populations would behave in a certain manner that could be predicted through the proxy of day-degrees accumulation. However, the PETE model for pesticide susceptible populations may not accurately predict the phenology of pesticide resistant CM populations, and therefore such models may need to be adjusted accordingly over the time.

In another study, we found conspicuous patterns of higher percent mortality (i.e., more insecticide susceptibility) for male CM collected from abandoned than commercial orchards for both first and second-generation flights in Pennsylvania (Joshi et al. unpublished data), indicating that CM populations in some Pennsylvania commercial apple orchards have developed some level of resistance toward azinphosmethyl, the primary insecticide of choice since the 1960s (Hull L.A., personal comm.). These insecticide resistant populations may develop at different growth rates, as the development of pesticide resistance may cause abnormalities in different life-stages and the life history of CM populations in commercial orchards. Such biological differences could be due to either an increase or decrease in the developmental growth rate by different life stages. Lue (2005) found a 9.8% increase in the egg development time of an organophosphate resistant population of CM, while Knight (2004) reported a delay in the spring emergence of CM and a positive correlation with azinphosmethyl tolerance. Such changes in the developmental time of insecticide resistant populations may alter adult CM emergence and flight patterns over the course of a season in commercial orchards, and this phenomenon may also explain some of the differences found in CM flight and adult emergence patterns in commercial orchard ecosystems.

In this study, the female PCM model predicted a delay in emergence at the initial 3–5% cumulative moth emergence period. This longer delay in the emergence of female moths vs. male moths could be due to protandry. A number of studies have reported that male CM emerge earlier than female moths (Newcomer and Whitecomb, 1924; Tadic, 1957; Putman, 1963), and this protandrous emergence behavior may be as early as 7–12 days before the start of female moth emergence (MacLellan, 1976).

The unconstrained and constrained PCM flight models described in this study are generated with a process-based deterministic logistic model approach. The major advantage of this approach is that it can very precisely predict CM phenology in an orchard that is closely related (particularly in terms of agronomic/cultural orchard practices, topography, pest management practices, and abundance and propensity of CM populations) to the orchards from which the initial biological parameters (e.g., moth capture, oviposition, and egg hatch) were obtained to specify the model. Therefore, applications of CM models (i.e., PCM constrained, unconstrained, and PETE models) based on this approach could be very effective in orchards of a specific type. However, the major weakness of such process-based models is that they ignore the uncertainty associated with CM flight patterns, which could encompass factors such as measurement errors, variable climatic conditions, and site/pest management program effects (Joshi et al., 2011b). Application of other modeling approaches (Damos et al., 2011) may be useful in such scenarios.

It is important to note that the PCM constrained and unconstrained models are derived from the cumulative percent moth capture using either the CM L2 or CM DAC lure baited traps in commercial orchards under conventional pesticide spray programs. Therefore, the unconstrained model may not perform well in orchards under sex pheromone mating disruption and other sex pheromone-treated scenarios. Being a deterministic process-based model, it does not consider variability due to the use of sex pheromone mating disruption in apple orchards. Besides the influence of pest management program type, the fit of these models may be significantly influenced by biofix (i.e., 1st sustained moth capture in traps) date. The accuracy of the biofix date at the beginning of moth flight may influence the model prediction performance by either overestimating or underestimating moth flight phenology over the entire season. However, such error in establishing the biofix could be eliminated by using highly attractive lures and monitoring systems, such as those utilized in this study. In both orchard types, two types of commercial lures were used to catch/monitor CM adults, and both types of lures baited traps were checked twice a week, and all the traps were rotated from one location to another to reduce any “hot-spot” based bias (Brunner, 2003). As a result, the error associated with moth capture in the traps was minimized, and both types of lure-baited traps yielded a very similar pattern of moth emergence from the beginning of flight season. However, such error associated with establishing biofix, low moth populations and poor moth capture can be eliminated by starting the accumulation of degree-days from 1 January of each year (Jones et al., 2008). Other aspects such as, variation in a localized strain of CM, level of pesticide resistance, growers' practices, apple cultivars, etc., may also directly or indirectly affect population abundance of CM in apple orchards, and over the time, such factors may significantly alter the completion time of an individual generation of CM. Consequently, all such aspects associated with apple production system may restrict the relative fit and performance of these models.

Studying comparative flight patterns of CM in commercial and abandoned apple orchard ecosystems may reveal some vital information related to the impacts of commercial apple production systems and pest management practices adopted over the past few decades on CM phenology. Applying comparative phenology models (moth capture based on either CM L2 or CM DAC lures) to commercial apple orchards under conventional pesticide programs in Pennsylvania should improve our understanding of CM flights, particularly the puzzling extended emergence and asymmetric flight peaks patterns. Furthermore, if correlated with CM egg hatch timings, the model predictions from the unconstrained CM flight model may also be very helpful in the pesticide spray decision-making process.

## Author contributions

NJ, ER, LH and GK designed the experiment. NJ and LH contributed in field work. NJ, LH, KN and ER contributed in data analysis and modeling. NJ prepared the manuscript with contributions from ER, LH, KN and GK. All authors reviewed the manuscript draft.

### Conflict of interest statement

The authors declare that the research was conducted in the absence of any commercial or financial relationships that could be construed as a potential conflict of interest.
